# BerryPortraits: Phenotyping Of Ripening Traits in cranberry (*Vaccinium macrocarpon* Ait.) with YOLOv8

**DOI:** 10.1186/s13007-024-01285-1

**Published:** 2024-11-13

**Authors:** Jenyne Loarca, Tyr Wiesner-Hanks, Hector Lopez-Moreno, Andrew F. Maule, Michael Liou, Maria Alejandra Torres-Meraz, Luis Diaz-Garcia, Jennifer Johnson-Cicalese, Jeffrey Neyhart, James Polashock, Gina M. Sideli, Christopher F. Strock, Craig T. Beil, Moira J. Sheehan, Massimo Iorizzo, Amaya Atucha, Juan Zalapa

**Affiliations:** 1 Department of Plant and Agroecosystem Sciences, University of Wisconsin-Madison, Madison, WI USA; 2 United States Department of Agriculture-Agricultural Research Service, Vegetable Crops Research Unit, Madison, WI USA; 3 Cornell University-Breeding Insight, Ithaca, NY USA; 4 Department of Statistics, University of Wisconsin-Madison, Madison, WI USA; 5 Department of Viticulture and Enology, University of California-Davis, Davis, CA USA; 6 Phillip E. Marucci Center for Blueberry and Cranberry Research & Extension, Chatsworth, NJ USA; 7 United States Department of Agriculture-Agricultural Research Service, Genetic Improvement of Fruits & Vegetables Laboratory, Beltsville, MD USA; 8 Rutgers University-Department of Plant Biology, New Brunswick, NJ USA; 9 Department of Horticultural Science, North Carolina State University, Raleigh, NC USA; 10 Plant Human Health Institute, North Carolina State University, Raleigh, NC USA

**Keywords:** Computer vision, Digital phenotyping, Image-based phenotyping, Image segmentation, Plant breeding, Pomology, Fruit quality

## Abstract

BerryPortraits (Phenotyping of Ripening Traits) is open source Python-based image-analysis software that rapidly detects and segments berries and extracts morphometric data on fruit quality traits such as berry color, size, shape, and uniformity. Utilizing the YOLOv8 framework and community-developed, actively-maintained Python libraries such as OpenCV, BerryPortraits software was trained on 512 postharvest images (taken under controlled lighting conditions) of phenotypically diverse cranberry populations (*Vaccinium macrocarpon* Ait.) from the two largest public cranberry breeding programs in the U.S. The implementation of CIELAB, an intuitive and perceptually uniform color space, enables differentiation between berry color and berry brightness, which are confounded in classic RGB color channel measurements. Furthermore, computer vision enables precise and quantifiable color phenotyping, thus facilitating inclusion of researchers and data analysts with color vision deficiency. BerryPortraits is a phenotyping tool for researchers in plant breeding, plant genetics, horticulture, food science, plant physiology, plant pathology, and related fields. BerryPortraits has strong potential applications for other specialty crops such as blueberry, lingonberry, caneberry, grape, and more. As an open source phenotyping tool based on widely-used python libraries, BerryPortraits allows anyone to use, fork, modify, optimize, and embed this software into other tools or pipelines.

**Supplementary Information:**

The online version contains supplementary material available at 10.1186/s13007-024-01285-1.

## Background

The evolution of human response to visual cues is an outcome of a commensurate evolutionary relationship between humans, seeking sustenance, and fruit, whose seeds are subsequently dispersed [1]. Whether due to natural selection or human-mediated selection, the plethora of fruit forms and hues are an avid area of research by horticulturists, plant geneticists, plant breeders, and food scientists [2]. However, measuring fruit characteristics in a high throughput manner has long been recognized as a major bottleneck in crop research [3]. For example, traditional measures of fruit color rely on categorical color bins, established by eye, in specialty crops such as plum [4], apricot [5], and eggplant [6]. Furthermore, many of the fruit quality traits that contribute to the hedonic human experience of fruit, such as color, are difficult to measure precisely. Genetic variation in photoreceptors among humans results in color perceptual differences; even humans without color deficient vision vary considerably in perceptual judgments of color due to variations in rod/cone receptors [7]. Thus, color estimations that rely on human perception reduce data accuracy, especially when differentiating between similar hues. In berry crops such as cranberry, grape, lingonberry, or blueberry, fruit color can be quantified with spectrophotometric measurements of the berry’s total anthocyanin (TAcy) pigments that humans perceive as red, purple, or blue, respectively. TAcy quantity is a continuous trait and permits finer color granularity than categorical phenotyping, but in cranberry and other berry crops, such assays are costly, laborious, destructive to fruit samples, and require specialized equipment and training [8, 9]. While fruit size and shape characteristics in berry crops such as cranberry, are generally less subjective attributes to quantify, these traits are also tedious, time-consuming, laborious, and costly to measure manually [10]. Traditionally, fruit length and width are measured by hand with calipers, and size metrics, such as surface area and volume, are calculated from these measurements [11]. This process requires substantial time and labor, especially when a large number of fruits are required to accurately characterize one population [12, 13].

The development of automated, high throughput image-based methods for crop phenotyping was motivated by the substantial cost, time, tedium, and difficulty of hand-measuring, estimating, or assaying these important quantitative traits [14, 15].

Computer vision has been implemented for phenotyping visual aspects of quality, ripeness, and harvestability with varying levels of throughput and accuracy in horticultural crops (Supp. table 1). Field-based imaging, or imaging fruit on the plants under natural lighting conditions, has been developed under various crop production environment (open field, greenhouse, orchard, vineyard) in a variety of specialty crops, such as, pumpkin [16], tomato [17, 18], pepper [19], and including perennial fruit crops such as citrus [20], quince and raspberry [21], blueberry [22–24], grape [25–28], myriad other horticultural specialty crops [29]. Comparison among these methods has recently been reviewed [30].

High throughput phenotyping methods are critical tools to advance research with accuracy and efficiency, and suitability of the platform varies depending on the crop, trait, and imaging environment [30]. Imaging fruit under natural lighting conditions can reduce color image quality due to variable daylight conditions. Fruit size distortion can occur due to the variable distances between the camera lens and fruits on the plant. Similarly, angular distortion can alter perception of fruit shape for non-spherical fruit, which occurs with variable angles of fruit on the plant relative to the lens of the camera. Finally, in the field, plant architecture and plant foliage may obscure imaging of the fruit, resulting in information loss due to occlusion [31]—at the time of publication, field imaging methods are still under development to impute or predict characteristics of obscured fruit in strawberry [31].

Postharvest fruit imaging in the laboratory mitigates all of the above issues by creating an environment with standardized lighting conditions, enabling repeatable and highly precise measurement of fruit quality traits. These types of standard conditions have been used in digital phenotyping pipelines for lettuce [33], carrot root and shoot [34, 35], tomato, winter squash [36], and in a variety of other specialty crops [37], including perennial fruit crops such as apple [38–40], cranberry [41], pear [40], blackberry [42], raspberry [43], strawberry [44–48], blueberry [49–52], and grape [27, 53]. Imaging on a stage, with all fruit at a fixed distance from the camera lens, eliminates fruit size distortion, while standard berry orientation eliminates fruit shape angular distortion.

Though there are numerous tools for other crops, there are very limited options for specialty crops such as cranberries. GiNA was the first high throughput phenotyping software program developed for postharvest imaging of cranberries under controlled lighting conditions [41]. This software was validated by measuring cranberries with a hand-held caliper and demonstrated very high correlations (0.92 ≤ r ≤ 0.97) between hand measurement and GiNA’s image-based measurement. GiNA has been proven effective for accurately phenotyping fruit color and has been leveraged as a high throughput image-based phenotyping tool in cranberry [8, 10], blueberry [54, 55], *Silphium integrifolium* [56], and kura clover (*Trifolium ambiguum*) [57].

Despite a track record of successful utilization, GiNA has high barriers to entry for users. GiNA runs on MATLAB (highly specialized and costly proprietary software), is unmaintained, and underperforms when cranberry shape deviates from a perfect ellipse, such as with pyriform, spindle, or ovate berries [58]. GiNA was validated on a small sample of fruit from one uniformly dark red commercial cultivar, which means that the software’s performance has not been tested on lighter berry colors that are abundant in diverse germplasm (e.g., green, yellow, white, and pink berries). These drawbacks required the creation of open source, user-friendly berry phenotyping software and subsequent validation on a larger and more diverse cranberry population.

Here we present BerryPortraits: Phenotyping Of Ripening Traits [‘with Rapid Automated Imaging Tools and Software’, for those disinclined towards brevity]) (https://github.com/Breeding-Insight/BerryPortraits/). This project is a collaboration with Breeding Insight, a USDA-ARS funded effort that works with identified speciality crops, providing support and development of phenotyping and genotyping tools. As the software name suggests, *BerryPortraits* measures the visual aspects of fruit quality and ripeness—berry color, size, shape, and uniformity—that are targets for selection in cranberry breeding programs. As a scalable, affordable, and high throughput phenotyping pipeline, BerryPortraits can extract berry data from images for color, size, and shape traits measured by GiNA. Trait accuracy can be measured by comparing BerryPortraits performance to its validated predecessor, GiNA. We also introduce BerryPortraits’s additional functionality of the CIELAB color space and improved calculations for measuring size characteristics such as volume and surface area. To improve accessibility and lower the barrier to entry for new users, BerryPortraits is open-source and was developed in Python, which is widely used for data science and has strong support with an active community of users troubleshooting problems in web-based forums. This pipeline also has the capacity to integrate with BreedBase [59] and the Android app Field Book [60], enabling precise traceability of phenotypic data and reducing probability of data loss or collection errors. With improved accessibility, affordability, throughput, and accuracy, BerryPortraits has innumerable possible creative applications for researchers in horticulture, plant genetics, plant breeding, and food science.

## Materials & methods

### Plant materials

The cranberry population under study, CNJ02-1, is the most well-studied diploid cranberry population available for genetic research [8, 9, 61–65]. This population was developed at Rutgers University, Chatsworth, NJ, by Nicholi Vorsa and consists of 171 cranberry genotypes representing a wide range of phenotypic variation in external fruit quality characteristics. Propagules from each genotype were clonally propagated and established at a commercial cranberry farm in Necedah, Wisconsin. Each genotype was grown in a distinct 2.25 m^2^ plot [61, 62] in beds that were professionally and uniformly managed. In September 2019, 30 mature cranberry fruit were randomly harvested from each plot and placed in mesh bags. Samples were placed in a styrofoam cooler and transported to the USDA-ARS Vegetable Crops Research Unit at the University of Wisconsin-Madison. Berry samples were maintained at 10 °C until ready for imaging.

### Berry image acquisition

Berries were photographed in an enclosed light box for controlled and standard lighting conditions (Supp. Figure 1). Images were taken with a DSLR camera (Canon EOS REBEL T6; focal length = 28 mm; F-stop = 11; exposure length = 1/20 s) with lens positioned directly over the fruit, approximately 70 cm above the stage. A sample of 30 cranberries from each plot were arranged on the stage in rows and columns of 5 × 6 berries (Supp. Figure 2) (this feature can be enabled or disabled in BerryPortraits). The stage background was white and included six 2.54-cm diameter black scale markers on either side of the fruit. One image was taken per 30-fruit sample for 171 unique genotypes (25–30 cranberries per image; image dimensions = 5184 × 3456 pixels in JPG format). Each image was evaluated in GiNA and in BerryPortraits for comparison of berry size, berry shape, and berry color values. Photo sample files were manually labeled with genotype ID.

For the purposes of training the instance segmentation model that underlies BerryPortraits, two other image sets were used in order to make the model more robust against variations in lighting, background, layout, and any non-target objects present in the image. The first set consisted of 281 images captured in the same manner described above, with three core differences: the image background was blue instead of white to increase contrast between the background and unripe berries; each image contained an X-Rite ColorChecker Classic card and a small human-readable card; and only six size markers were used instead of twelve. The second set consisted of 60 images (25–50 berries per image) captured on white or speckled gray-black divot trays with a wide range of berry sizes, image layouts, and presence and position of size and/or color markers (personal communication—Jeff Neyhart). For representative examples of each image type, see Supp. Figure 3.

### Running BerryPortraits

The core output of BerryPortraits is a.csv file with metrics for each detected berry in each input image, similar to the output of GiNA. If berries are arranged into rows and columns, BerryPortraits can auto-detect the number of rows, assign berries a row and column, and organize the output by row/column. If berries are not organized, the output values can be tied to a given berry in the image using the pixel coordinates of the berry centroid.

For visualization and quality control, BerryPortraits can save annotated versions of each input image showing the berry mask boundaries, size marker mask boundaries, and oriented bounding boxes (OBBs) around each berry. Examining these annotated images by hand is a fast way to check that the model is detecting and segmenting objects correctly, and we recommend users do so as a matter of course. Several output values are checked during runtime as a basic QC. Any berries with solidity < 0.95 (see below for explanation) are flagged and reported, as these are typically berries with incorrect segmentation. Any berries with an area > 3 standard deviations from the mean area of the image are similarly flagged.

### BerryPortraits object identification and segmentation

The first step of the BerryPortraits analysis pipeline is the detection and segmentation of berries and size markers. The default method uses YOLOv8 [66], a widely-used state of the art (SOTA) model (at the time of publication, cited more than 12,700 times) for detection and instance segmentation of four object classes: cranberries, black circular size markers (2.54-cm diameter in the image sets here), sample labels (black text on white cards), and X-Rite ColorChecker Classic color calibration cards. The YOLOv8 architecture was selected because it performed well on validation images and it offered pretrained instance segmentation models, unlike pure detection models such as YOLOv9 [67], YOLOv10 [68], or YOLO-NAS [69], which must be combined with downstream segmentation methods. This single-step instance segmentation greatly simplified training the model, deploying the script, and retraining the model on additional data. Meta’s Segment Anything Model (SAM) [70], a general-purpose instance segmentation model, was tested during development and performed well, but the large sizes of SAM model files (358 MB to 2.56 GB) made it a less attractive choice than YOLOv8 in terms of distributing this tool.

The dataset used for training, validation, and testing consisted of 512 images: 171 white-background images and 60 variable-layout images (described above under *Image Acquisition*), and 281 blue-background images (25 berries per image). Images were annotated with polygon borders around objects in the four target classes and partitioned into a 70/20/10 split between training/validation/test. A pretrained YOLOv8s-seg base model was then trained for 100 epochs on an NVidia A100 with single-image batch size at an image resolution of 3200 using hyperparameters set automatically by the Ultralytics library based on the image and annotation metadata.

We also included a traditional computer vision method for detecting and segmenting cranberries and size markers using OpenCV. This approach uses several static thresholding steps on the hue, saturation, and value channels and several. This method is more sensitive to non-target objects and variation in background or object layout, but is extremely fast and reliable for images laid out on a clean background in a consistent manner with no non-target objects.

Because GiNA does not organize berries into rows and columns, we used the pixel coordinates of berry centroids to match berries 1-to-1 in order to compare the size and color metrics reported below between GiNA and BerryPortraits. For a given image, the Euclidean distance between each pair of berry centroids from GiNA and BerryPortraits were calculated and berries were considered the same if they were the reciprocally closest berries to one another.

### BerryPortraits size and shape metrics

Size metrics of detected berries are calculated from the segmentation mask around the berry and are reported in pixels by default or are dimensionless. If the user has included circular size markers of a known diameter, this is used to adjust size metrics to cm. *Length* and *widt*h of each berry are defined by the length and width of the oriented bounding box (OBB) surrounding the berry, the direction of which is taken from the primary axis of the ellipse of best fit, thus berries do not have to be oriented before measuring (Table 1). This method attempts to match the length and width measurement that would be taken with a hand-held caliper.

**Table 1:** Shape and size formulas and descriptions used by GiNA vs. BerryPortraits

Trait	GiNA description/formula	BerryPortraits description/formula
Length	Length of the major axis that has the same normalized second central moments as the region. It is always larger than the width, therefore this parameter will be confounded in objects that are wider than they are long	Length of the oriented bounding box (OBB) around the berry segmentation mask, oriented along the primary/major axis of the ellipse of best fit
Width	Length of the minor axis that has the same normalized second central moments as the region. It is always shorter than the length	Width of the OBB around the berry segmentation mask, orthogonal to the primary/major axis
Area	Number of squared units in the recognized object. The same pixels counter in this parameters are used to determine color	Area of the berry segmentation mask
Surface area	Estimation of the surface area assuming that the object is spherical or egg shaped $2\pi {a}^{2}+\mu a*2(\frac{{b}^{2}}{\sqrt{{b}^{2}-{a}^{2}}}\frac{a}{b} )$ where $a$ and $b$ are 0.5*width and 0.5*length, respectively Note: GiNA software inexplicably or erroneously multiplied this formula by a factor of 50	Approximated as the sum of lateral areas of a series of stacked truncated cones, each with an axis 1 pixel high on the major axis of the OBB and base/top widths equal to the width of the berry mask, orthogonal to the major axis, at given pixel heights. Base and top area are added only for the first and last truncated cone: $\pi [{ (\frac{{w}_{1}}{2})}^{2} +{ (\frac{{w}_{h}}{2})}^{2} + {\sum }_{i=1}^{h-1}{ (\frac{{w}_{i}}{2}}+{ \frac{{w}_{i+1}}{2})}\sqrt{{ (\frac{{w}_{i}}{2}}-{ \frac{{w}_{i+1}}{2})}^{2} + 1)}$ where ${w}_{1}$ = width at the mask base, ${w}_{h}$ = width at the top of of the mask, ${w}_{i}$ = mask width at a given pixel height $i$, and $h$ = total height in pixels of the mask
Perimeter	Distance around the boundary region of the object. It is computed by calculating the distance between each adjoining pair of pixels around the border of the region	Arc length of the segmentation mask
Volume	Estimation of the object volume assuming that the object is spherical or egg shaped $\frac{2\pi }{3}{a}^{2}(a+b)$, where $a$ and $b$ are 0.5*width and 0.5*length, respectively Note: GiNA software inexplicably or erroneously multiplied this formula by a factor of 4	Approximated as the sum of volumes of a series of stacked cylinders, each with an axis 1 pixel high on the major axis of the OBB and a width equal to that of the berry mask, orthogonal to the major axis, at a given pixel height: ${\sum }_{i=1}^{h-1}{ (\frac{{w}_{i}}{2})}^{2}$, where $h$ = total height in pixels of the mask and ${w}_{i}$ = width in pixels of the mask at pixel height $i$
Shape/Roundness	Describe how round is the object. A value of 1 represents a circle-like object Length: width	ISO 110 Roundness definition: $\frac{{r}_{inscribed}}{{r}_{enclosing}}$, Where ${r}_{inscribed}$ = radius of the largest circle inscribed by the berry segmentation mask and ${r}_{enclosing}$ = radius of the smallest circle circumscribing the berry mask
Solidity	-	$\frac{{A}_{mask}}{{A}_{hull}}$, Where ${A}_{mask}$ = area of the berry mask and ${A}_{hull}$ = area of the convex hull circumscribing the mask

GiNA description column reprinted with permission from Table S1, Diaz-Garcia et al. [41]: Description of the parameters generated by GiNA. Mathematical formulae and description of parameters returned by the software

*Perimeter* and *area* are defined as the arc length and area of the segmentation mask. *Solidity* is defined as the ratio of the area of the segmentation mask to the area of the convex hull around the mask. *Roundness* is defined using the ISO 110 definition: the ratio of the radius of the largest circle inscribed by the segmentation mask to that of the smallest circle inscribing the mask. In contrast to GiNA, BerryPortraits estimates surface area and volume by modeling the berry as a series of stacked cylinders. Volume is approximated by modeling the berry as a series of stacked cylinders with diameter equal to the segmentation mask width and a height of 1 pixel. Similarly, surface area is approximated by modeling the berry as a series of stacked frustums (truncated cones) with upper and lower diameters taken from the segmentation mask at pixel height *h* and height *h* + *1* (Supp. Figure 4). Testing of these traits on ellipsoids drawn at different pixel resolutions found the volume estimation to be within 0.05% of the true value and the surface area approximation to be within 5% of the standard approximation (as the surface area of an ellipsoid has no closed-form solution).

We identified formulaic discrepancies in GiNA that inexplicably or erroneously multiplied surface area and volume values by a factor of 4 and 50, respectively. For our analyses, we performed a transformation on the GiNA output to enable comparison. The formulae used for each platform are reported (Table 1).

### BerryPortraits color metrics

As illumination and camera settings can differ between image sets, BerryPortraits uses the ColorCorrectionML library (https://github.com/collinswakholi/ML_ColorCorrection_tool) to perform post hoc color correction using linear correction, least-squares regression, or partial least-squares regression. At the time of writing, ColorCorrectionML can correct images captured with a standard D50 illuminant and that contain an X-Rite ColorChecker Classic. Color correction is disabled by default.

Berries with considerable epicuticular waxes tend to strongly reflect the background surface along the edge of the berry, distorting color metrics. To mitigate this, BerryPortrait erodes a 10-pixel border off of the edge of each berry before calculating the mean and standard deviation of the L*a*b* and RGB color channels.

We were interested in comparing the outputs for color traits measured by both GiNA and BerryPortraits. Both platforms evaluate berries using all three RGB color channels, which were then used to calculate grayscale (Table 2).

**Table 2:** Color formulae used by GiNA and BerryPortraits

**Trait**	GiNA [41]	BerryPortraits
RGB-color	Arithmetic mean and median of red, green, and blue color channel values, considering all the pixels in the detected object	Same as GiNA
Grayscale	A transformation of the RGB to a gray-color space: Red_median * 0.299 + Blue_median * 0.114 + Green_median *0.587	Same as GiNA
Color variation	Variance of the RGB color and grayscale color across all the pixels in the object	Same as GiNA
L*	–	Perceptual lightness Scale: 0 (black)—100 (white) From CIELAB
a*	–	Green (−)/Red (+) color axis From CIELAB
b*	–	Blue (−)/Yellow (+) color axis From CIELAB

Description of parameters returned by the software

We also developed additional color metrics that were not readily available in GiNA. Defined by the International Commission on Illumination *(CIE: Commission Internationale de l’Eclairage*), the CIELAB color space is a perceptually uniform color space that expresses color as three values based on human color perception: L* for luminance (perceptual lightness), a* for red/green color coordinates, and b* for blue/yellow color coordinates—four unique colors of human vision from the visible spectrum [71]. Because these features are not available in GiNA, no comparison between platforms was possible for these traits.

### Statistical analyses

We were interested in all pairwise comparisons of geometric and color traits measured by both GiNA and BerryPortraits. All analyses were performed in R (version 4.3.1) [72] with assistance from the *tidyverse* suite of packages [73]. Two-sample paired t-tests were performed for all traits between platforms. We quantify trait preservation (Tables 3 & 4) with mean trait difference between platforms, normalized by the smaller range of trait values within a single platform—we refer to this effect size as *range-normalized mean difference* (rmd) defined as the following with $\overline{d}_{t}$ as the mean difference for trait (t), ${x}_{(n)}, {x}_{(1)}, {y}_{(n)},{y}_{(1)}$ as the max and min of BerryPortraits (*x*) and GiNA (*y*) respectively:

$${\text{rmd}}_{t} = \frac{{\overline{d}_{t} }}{{\min \left( {x_{\left( n \right)} - x_{\left( 1 \right)} ,y_{\left( n \right)} - y_{\left( 1 \right)} } \right)}}$$

**Table 3:** For each trait and each platform: comparison of population means, population minimum values, population maximum values, lower confidence intervals (LCI), upper confidence intervals (UCI), mean differences (md), range-normalized mean differences (rmd), and Pearson correlation coefficients (r) among shape and size parameters comparing BerryPortraits and GiNA

Trait	Platform	Mean	Min	Max	LCI	UCI	md	rmd	r
Length (cm)	BerryPortraits	2.03	1.27	2.90	2.03	2.04	0.08	0.05	> 0.98
	GiNA	1.95	1.24	2.81	1.94	1.95			
Width (cm)	BerryPortraits	1.66	1.10	2.19	1.66	1.66	0.06	0.06	> 0.97
	GiNA	1.60	1.04	2.09	1.60	1.60			
Perimeter (cm)	BerryPortraits	6.19	3.98	8.50	6.17	6.20	0.63	0.14	> 0.97
	GiNA	5.56	3.54	7.40	5.55	5.58			
Area (cm^2^)	BerryPortraits	2.59	1.07	4.37	2.58	2.60	0.13	0.04	> 0.99
	GiNA	2.46	1.01	4.13	2.44	2.47			
Surface area (cm^2^)	BerryPortraits	9.94	4.15	16.78	9.89	9.98	0.25	0.02	> 0.93
	GiNA	9.69	3.94	17.13	9.64	9.74			
Volume (cm^3^)	BerryPortraits	2.91	0.78	6.24	2.89	2.93	1.5	0.29	> 0.99
	GiNA	1.33	0.35	2.85	1.32	1.34			

GiNA from Diaz-Garcia et al. [41]

BerryPortraits from present study, Loarca et al.

**Table 4:** For each trait and each platform across all images (N = 171): Comparison of population means, population minimum values, population maximum values, lower confidence intervals (LCI), upper confidence intervals (UCI), mean differences (md), range-normalized mean differences (rmd), and Pearson correlation coefficients (r) among color parameters comparing BerryPortraits and GiNA

Trait	Platform	Mean	Min	Max	LCI	UCI	md	rmd	r
Red channel (RGB)	BerryPortraits	95.65	26	188	94.66	96.63	1.95	0.01	> 0.99
	GiNA	97.59	29	189	96.64	98.55			
Green channel (RGB)	BerryPortraits	36.72	17	193	36.01	37.43	2.80	0.02	> 0.99
	GiNA	39.52	19	194	38.81	40.22			
Blue channel (RGB)	BerryPortraits	31.46	16	132	31.04	31.88	2.48	0.02	> 0.99
	GiNA	33.94	18	132	33.53	34.35			
Grayscale (RGB)	BerryPortraits	53.73	24	183	53.03	54.45	2.51	0.02	> 0.99
	GiNA	56.25	26	184	55.55	56.94			
L* (CIELAB)	BerryPortraits	60.34	21	195	59.56	61.13	–	–	–
	GiNA	–	–	–	–	–			
a* (CIELAB)	BerryPortraits	24.63	− 18	44	24.35	24.92	–	–	–
	GiNA	–	–	–	–	–			
b* (CIELAB)	BerryPortraits	16.37	0	51	16.15	16.60	–	–	–
	GiNA	–	–	–	–	–			

GiNA software from Diaz-Garcia et al. [41]

BerryPortraits software from present study, Loarca et al.

Since the magnitude of raw mean difference will be distorted by the scale of the trait, the chosen measure of effect size (rmd) aims to place all traits on a comparable scale so as to identify traits that are more variable. We normalize by the range within a single platform to emphasize that measurements from a single platform will likely vary more than a change between the platforms.

Pearson correlation coefficients were calculated to measure strength of correlation between both platforms for each trait. Correlation among traits in each platform was evaluated with a scatterplot matrix. Curvilinear regression lines were fit by local regression (LOESS) for each pair of traits for each platform. Means for each trait are reported with 95% confidence intervals for each platform. We also visually compared sample distributions between platforms for each trait with density plots and histograms. Overlapping density plots (similar means and variances) indicate substantially similar performance between platforms. Non-overlapping density plots, with larger mean differences or more unequal variances, indicate dissimilar trait performance between platforms.

## Results

### YOLOv8 model performance

The YOLOv8 model was able to detect and segment cranberries with high accuracy across a variety of backgrounds. On a pixel-wise segmentation mask classification basis, the precision (true positives/true positives + false positives) was 0.997 and the recall (true positives/true positives + false negatives) was 0.999, for a pixel-wise F1 value of 0.998. The mask mAP50 (area under the precision-recall curve averaged over all classes when an intersection-over-union of 0.5 is considered a true overlap) was 0.995. The mask mAP50-95 (mean area under the precision-recall curve across all classes, averaged over intersection-over-union values ranging from 0.5 to 0.95 in 0.05 increments) was 0.927.

The only task where the model consistently struggled was in correctly segmenting very light berries from a white background. In all instances that we could find, an incorrect berry segmentation mask was accompanied by a berry solidity < 0.95; given this, we added a flag at runtime notifying the user of any berry with solidity under this value.

### Platform comparison for shape and size traits

The output of each platform’s image analysis includes size and shape values for each berry in each image. Trait means, 95% confidence intervals, and range-normalized mean differences are provided for shape and size parameters (Table 1).

**Fig. 1:**
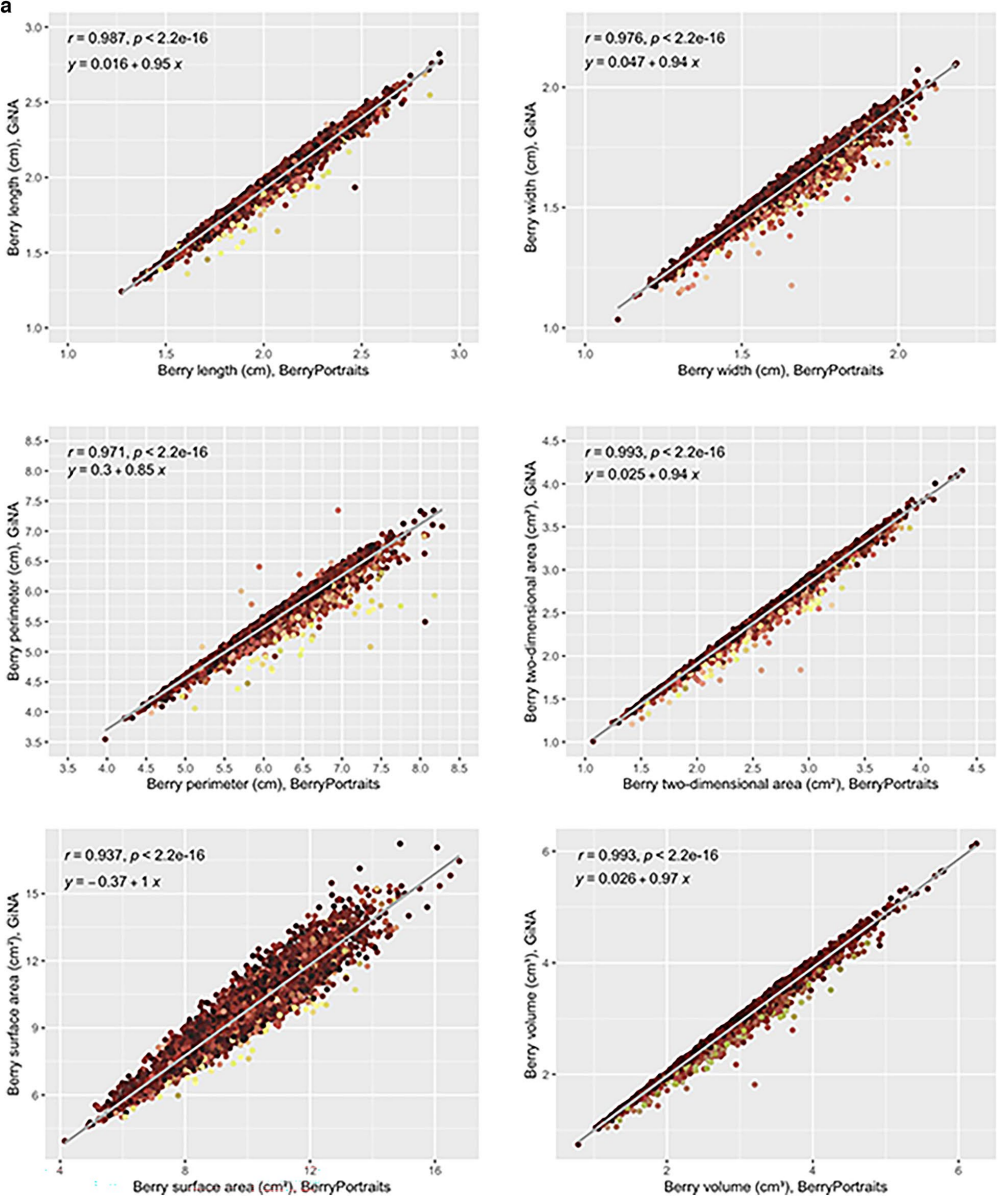

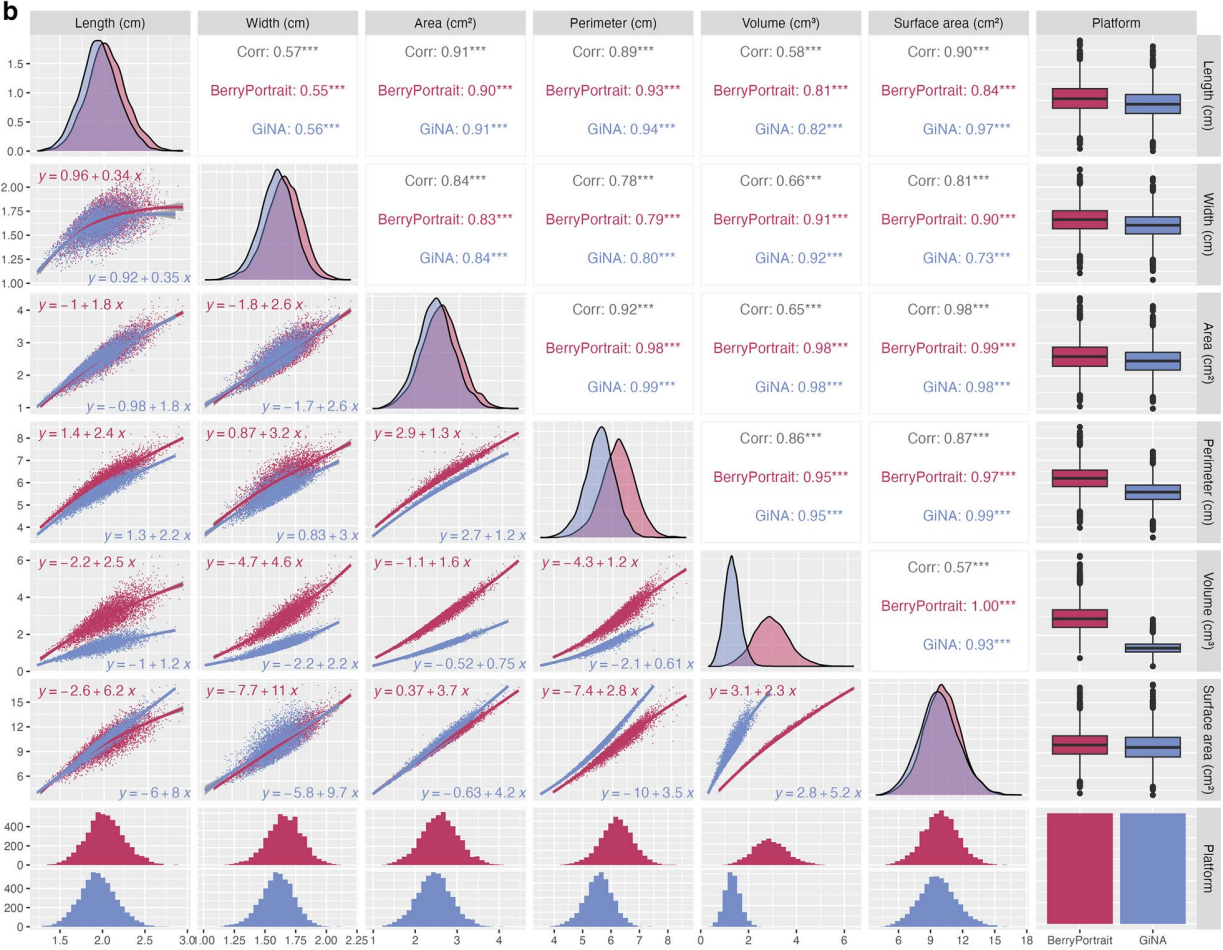
**a** Pearson’s correlation between size and shape parameters in BerryPortraits and GiNA, n = 171 samples. **b** Pearson correlation coefficients and single linear regression formulae among shape and size traits in BerryPortraits and GiNA. Shape and size relationships are generally conserved between platforms

Range-normalized mean differences (rmd) for shape and size traits were generally very low (0.02 ≤ rmd ≤ 0.06), except for perimeter (rmd = 0.14) and volume (rmd = 0.29). Correlation coefficients between platforms for shape and size traits were extremely high (0.97 ≤ r ≤ 0.99) to very high (r = 0.94 for surface area) (Fig. 1a). Due to the large sample size (N = 171 photos with 30 fruit per photo), all t-tests comparing trait performance between platforms yielded statistically significant results (p ≤ 0.05; not shown), though the estimated differences for many of the traits are too small to be of meaningful concern. Overlapping density plots (similar means and variances) indicate substantially similar performance between platforms for all shape and size traits except perimeter area and volume (Fig. 1b, diagonals). Regression equations for each trait pair were also highly similar between platforms (Fig. 1b, lower panels). Correlations among traits, factored by platform, typically differed by 0.0–0.02 (Fig. 1b, upper panels). The largest differences in correlations between platforms were for surface area x length (0.84 ≤ r ≤ 0.97), surface area x width (0.73 ≤ r ≤ 0.90), and volume (0.93 ≤ r ≤ 1). Coefficients of the regression equations for each trait pair were also highly similar between platforms, except for all relationships related to volume (Fig. 1b, lower panels). Comparing solidity between platforms, we found that BerryPortraits (3 berries) had fewer berries below the 0.95 threshold than GiNA (9 berries out 5130), indicating that, while both platforms demonstrate excellent ability to segment fruit, BerryPortraits has higher overall performance for solidity (Supp. Figure 5). There is no overlap between the berries that segmented poorly in either platform. Within the 0.95 quality threshold, segmenting ability in GiNA appears to decrease with pink or light red fruit, while in the same region for Berry Portraits, solidity appears to decrease with yellow fruit.

### Platform comparison for color traits

The output of each platform’s image analysis includes color trait values for each berry in each image. Color trait means, 95% confidence intervals, and range-normalized mean differences are provided (Table 4).

**Fig. 2:**
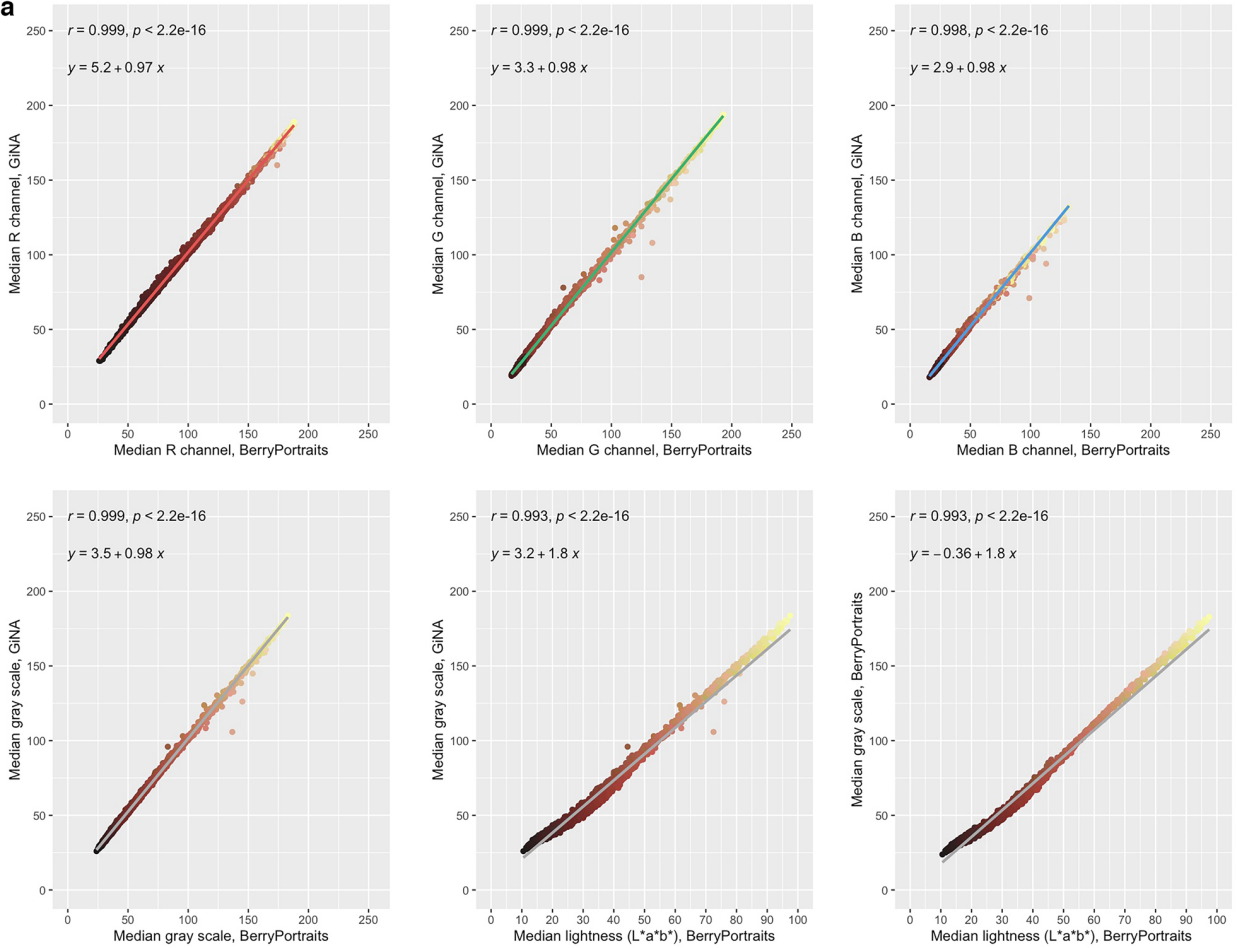

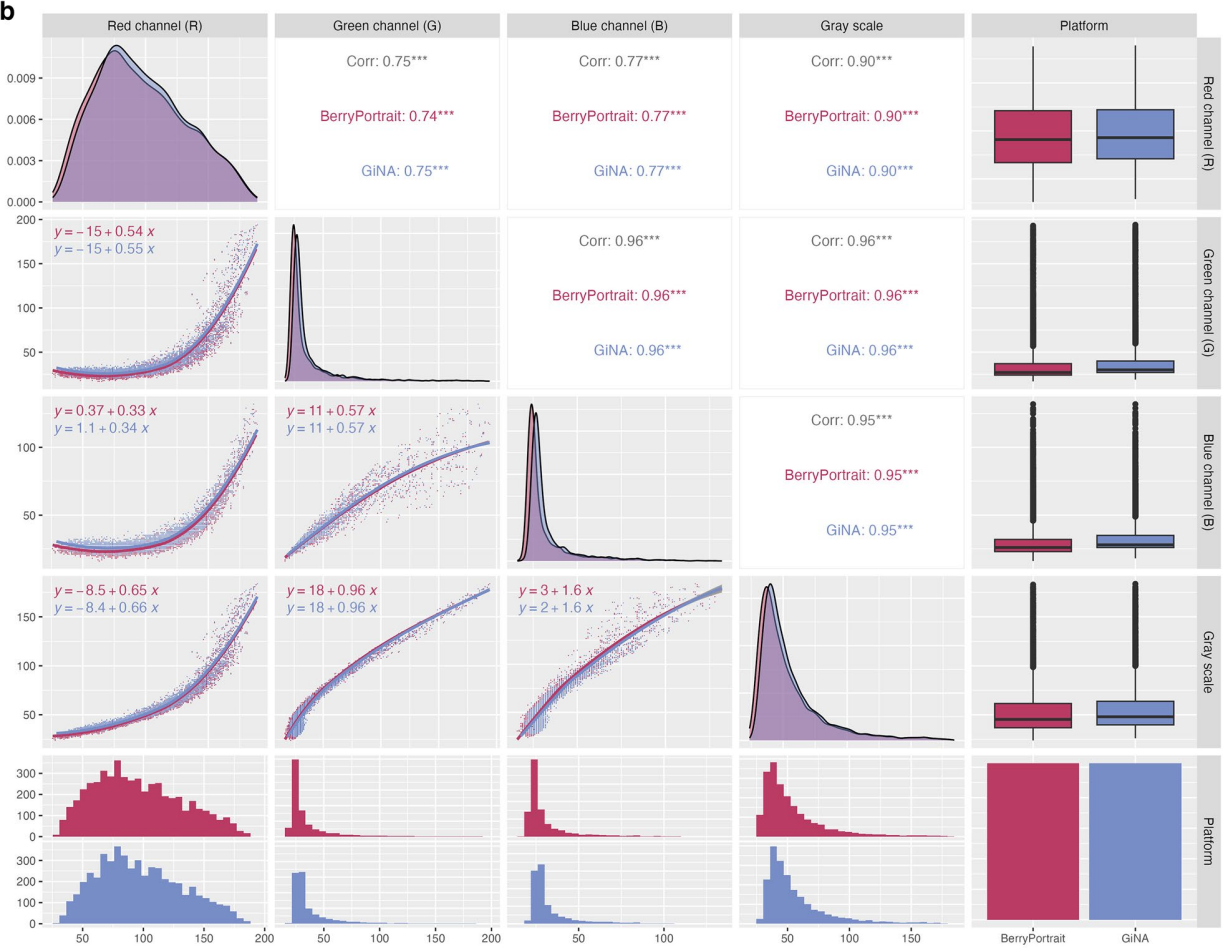
**a** Pearson’s correlation between RGB, grayscale, and lightness (L*a*b) values in BerryPortraits and GiNA. **b** Pearson correlation coefficients and single linear regression formulae among RGB and grayscale in BerryPortraits and GiNA. Color trait relationships are conserved between platforms

RGB and grayscale demonstrated very low range-normalized mean differences (0.01 ≤ rmd ≤ 0.02). Correlation coefficients between platforms for RGB were nearly identical (r > 0.99) (Fig. 2a—top row) and identical for grayscale calculated from RGB (r = 1) (Fig. 2a, bottom row). Luminance (L*, perceptual lightness) in BerryPortraits correlates almost identically (r > 0.99) with grayscale calculated from RGB values in both GiNA and BerryPortraits. Overlapping density plots (similar means and variances) indicate substantially similar performance between platforms for all color traits (Fig. 2b, diagonals). In RGB and grayscale, correlation coefficients among traits were highly similar for both platforms and density plots overlapped substantially for all traits (Fig. 2b, upper panels). Correlation coefficients differed by 0.00–0.01. Coefficients of the regression equations for each trait pair were also highly similar between platforms, except for red channel × blue channel—while the slopes were nearly identical, the intercepts varied between platforms (Fig. 2b, lower panels). We visualized output of the CIELAB color space by converting values to hex code. Each data point represents the average cranberry color from each berry in each photo (Supp. Figure 6) and enables rapid evaluation of the range of color variation of berries in our population.

## Discussion

BerryPortraits is an open source high throughput image-analysis pipeline for cranberry morphometric traits, making this software broadly applicable to horticultural research by food scientists, plant physiologists, plant breeders, and plant pathologists, and with strong potential for application in other fruit crops. We tested this software on cranberry populations from the two largest public cranberry breeding programs in the U.S., which makes this the largest cranberry image dataset ever to be validated. BerryPortraits has all the capacity to accurately measure digital images for all morphometric traits (length, width, area, volume, surface area, RGB, grayscale) measured by the previous unsupported, unimproved, and outdated software (GiNA), as well as new traits that were not available before, such as an intuitive color space (CIELAB and HSV), shape estimation improvements (surface area and volume), and uniformity metrics. BerryPortraits output enables rapid and accurate phenotyping for traits that require a large number of berries per genotype to accurately characterize, thus reducing the time and labor in phenotyping. Though the tool was originally conceived and designed for cranberry breeding populations, it has many potential applications for a wide range of plant science researchers to efficiently and accurately collect data on fruit quality traits.

### Platform performance

In this study, we are using correlation between BerryPortraits with the validated software, GiNA, as a measure of ground-truthing. In the original paper, GiNA was validated using correlation between red color in berry images and berry anthocyanin content (r = 0.93) [41]. BerryPortraits red channel correlated very highly with GiNA (r_R_ > 0.99). GiNA length and width measurements were validated by comparing hand caliper measurement of individual berries to length and width extracted from images (0.92 < r < 0.97), indicating very high accuracy. BerryPortraits length and width correlated very highly with GiNA’s measurements (r_L_ > 0.98; r_W_ > 0.97; Table 3).

### Platform methodology comparison

Both platforms offer classical computer vision and artificial neural network (ANN) approaches to perform semantic segmentation, a necessary preprocessing step needed to isolate foreground berry instances from extraneous background image components. However, the two platforms differ in the sophistication and capability of their ANNs to conduct segmentation.

BerryPortraits’ underlying neural network segmentation framework is based on YOLOv8, a widely used SOTA model. YOLOv8 is pre-trained on a set of components useful to the berry inference process, such as berries, color cards, a variety of scale marker types, and text labels. This pre-trained and validated YOLOv8 is subsequently used to detect pertinent objects, returning an inference confidence value, the predicted class label, and a bounding box. BerryPortraits subsequently calculates the center of the object’s bounding box and inputs the associated points and object class to a pre-trained model to extract a refined segmentation mask of relevant objects. This segmentation method balances classification performance with flexibility to accurately extract multi-class elements from an image containing berries. In contrast, GiNA uses a multilayer perceptron (MLP) with a single hidden layer to do simple binary classification of pixels into foreground berries or background elements. This MLP is trained using two RGB images: a foreground image containing a manual sample of representative colors of berry pixels and a background image containing characteristic background colors. This binary classifier is synonymous with classical computer vision segmentation approaches using thresholded colorspace channels, in that it lacks the fidelity to robustly incorporate and classify other useful objects in an imaging scene, such as a sample label, color card, and scale markers—a capability that is built in to BerryPortraits.

### Geometric features

BerryPortraits can quickly inspect outlines and oriented bounding boxes across dozens of images to visually confirm that the berry shapes as detected by BerryPortraits are correct. Length and width can easily be validated by hand, and even if the original fruits are no longer available, data can be compared to the typical values for berry size to confirm that it falls within the expected ranges.

Nearly one-to-one correlations between size and shape traits measured by BerryPortraits and GiNA (Fig. 1a) indicates a very strong relationship between two measurements. In most cases, corresponding traits that had high correlations between platforms also had means and variances that were substantially in agreement between platforms (diagonal of Fig. 1a—overlapping density plots). Therefore, BerryPortraits provides an equivalent estimate of length, width, area, and surface area, and can be used with confidence by researchers.

High correlation does not necessarily suggest equivalence. Though perimeter and volume are highly correlated between the GiNA and BerryPortraits (Table 3), density plots suggest a lack of agreement (Fig. 1a—non-overlapping density plots). This discrepancy is not surprising given that each platform had a different method for calculating volume. Berry volume and surface area are not easily measurable or ground-truthed with hand measurements—as such, these trait values were by necessity calculated from length and width. GiNA estimates surface area and volume by assuming a perfectly elliptical object, which does not work well for irregular shaped fruit (e.g., pyriform, spindle, or ovate shaped fruit). BerryPortraits has made substantial improvements compared with GiNA by addressing the technical challenge of measuring irregularly shaped fruit, by adjusting the method for calculating volume such that it is not limited by the assumption of perfect ellipses. We thus expect to produce more accurate volume measurements for imperfect ellipsoids and by extension to improve our ability to detect significant marker associations for shape and size characteristics in cranberry and other irregularly shaped fruit crops. It is noteworthy that GiNA’s role in the successful identification of three significant multi-year genetic markers (PVE < 10%) associated with image-based fruit size in blueberry—which correlates highly with fruit weight (r > 0.97), a very important factor in fruit quality [55]—lends credibility to the use of image-based fruit size in future fruit studies.

### Color metrics

**Fig. 3:**
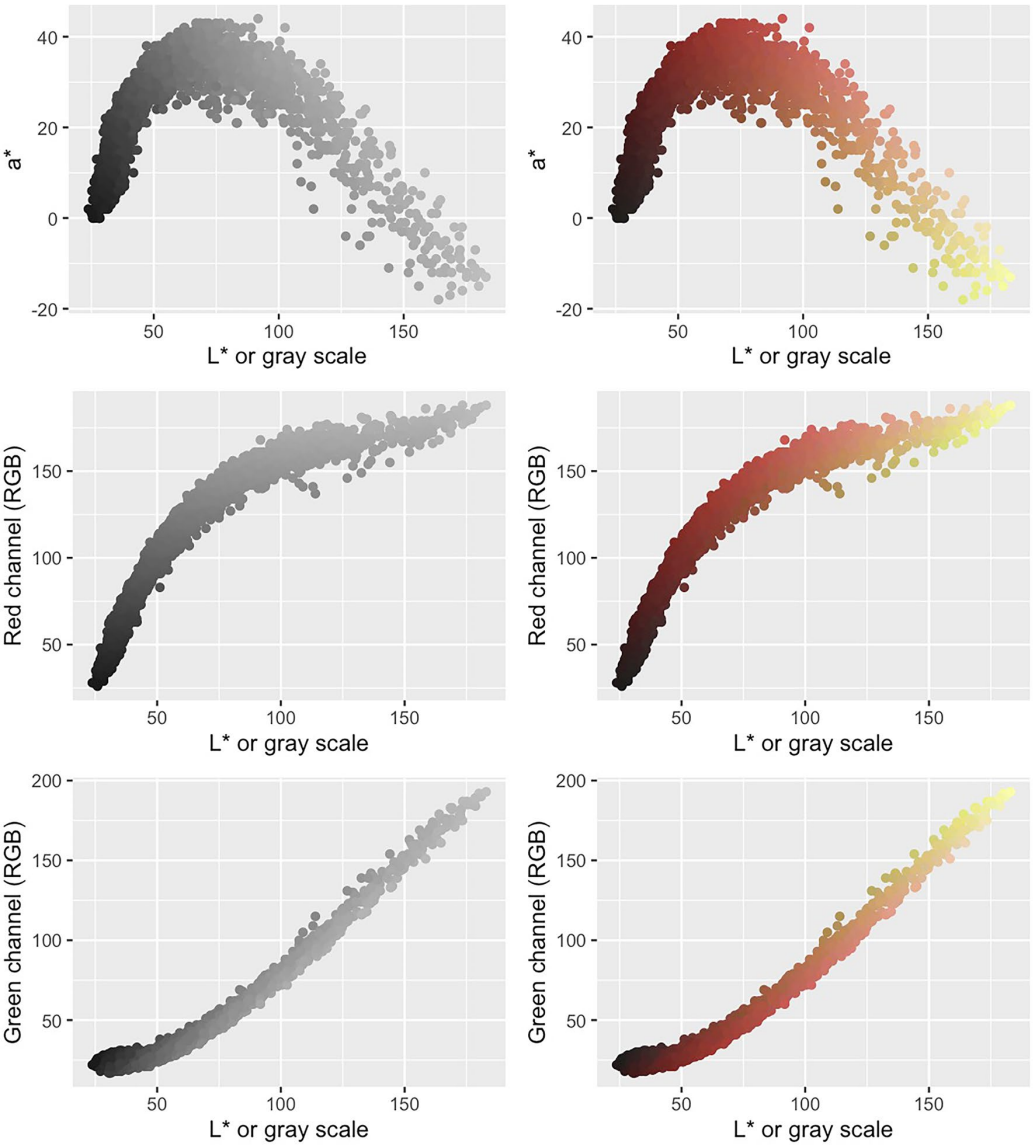
In RGB color space, red channel and green channel confound berry color intensity (y axis) with berry lightness or darkness (x-axis). Berries with intense red pigment are also perceived as darker, while yellow berries are perceived as lighter. In CIELAB color space, color intensity is independent of perceived lightness (L*). The y-axis (a*) represents the red-green color axis from the CIELAB color space

Since cranberries deepen in pigment dramatically as they ripen and accumulate anthocyanins, berry color can be a proxy for total anthocyanin content (TAcy), which is useful since anthocyanin quantification is costly and laborious. Color metrics do not need to be complex in order to be useful—for instance, mean berry grayscale values as measured by GiNA were correlated with TAcy (*r* = 0.83) [8]. Both grayscale and RGB values have limitations, however. Grayscale values cannot distinguish between shades of green and red with similar grayscale values, which are often seen during the middle stage of ripening. Differences in RGB channel values are not perceptually uniform; two color differences of the same numerical value may look very different in magnitude to the human eye [74]. GiNA only measures color with RGB, in which all three color channels are confounded with overall brightness—GiNA outputs grayscale values, but green/yellow (under-ripe) and red (ripe) berries can have very similar grayscale values (Fig. 3). The red channel (R) has counterintuitive color interpretation, as green fruit have highest R values and dark red fruit have lowest R values. A user selecting varieties with high mean R values would be selecting for those berries that are brightest, i.e. those that are least ripe and with the *lowest* TAcy.

While BerryPortraits reports RGB and HSV values for completeness, the primary color metrics intended for downstream use are per-berry values in the L*a*b* (also referred to as CIELAB) color space. This has several advantages over the RGB color space. L*a*b* separates color into a lightness axis L* and two color axes a* and b*, avoiding the issues of color channels being highly confounded with brightness (Fig. 3). The a* dimension represents a color’s position along roughly a red-green axis, making it an obvious a priori choice for fruits which change from red to green during ripening. Finally, the scale was designed so that differences in color would be perceptually uniform; if two objects differ in color, and their colors in L*a*b* coordinates when viewed under standardized conditions differ by a given Euclidean distance ${\Delta }_{E}$, this difference looks roughly the same to the human eye no matter in the color space the two objects lie [74]. Mean/median a* value on a per-berry basis is a useful proxy for complete color development for red berries. Mean/median L* is a simple proxy for overall berry brightness (ripeness): unripe berries are lighter in color and have higher L*, while ripe berries are darker in color and have a lower L*. It is highly correlated to grayscale value but offers slightly more resolution on darker berries. L* in isolation is not suited for identifying berries in an acceptable window along a ripeness gradient. A breeder looking to quickly summarize which fruits are fully ripe, but not overripe, could not do so using L* data alone. Low variance of image L* indicates synchronous, uniform brightness and ripening across berries (berries are similar in ripeness to one another), while low variance of individual berry L* indicates uniform individual berry color development. Low variance of image a* indicates synchronous, uniform color development across berries (berries are similar in color to one another), while low variance of individual berry a* indicates uniform individual berry color development.

We recommend that users rely on three metrics to understand the color of their imaged samples: mean or median L* as a proxy for sample brightness (ripeness) (stabilizing selection); standard deviation of L* as a proxy for uniform sample ripeness (low values); mean/median a* as a measure of sample redness (high values); and standard deviation of a* as a proxy for sample color uniformity (low values).

### Significance in berry crop breeding

**Fig. 4:**
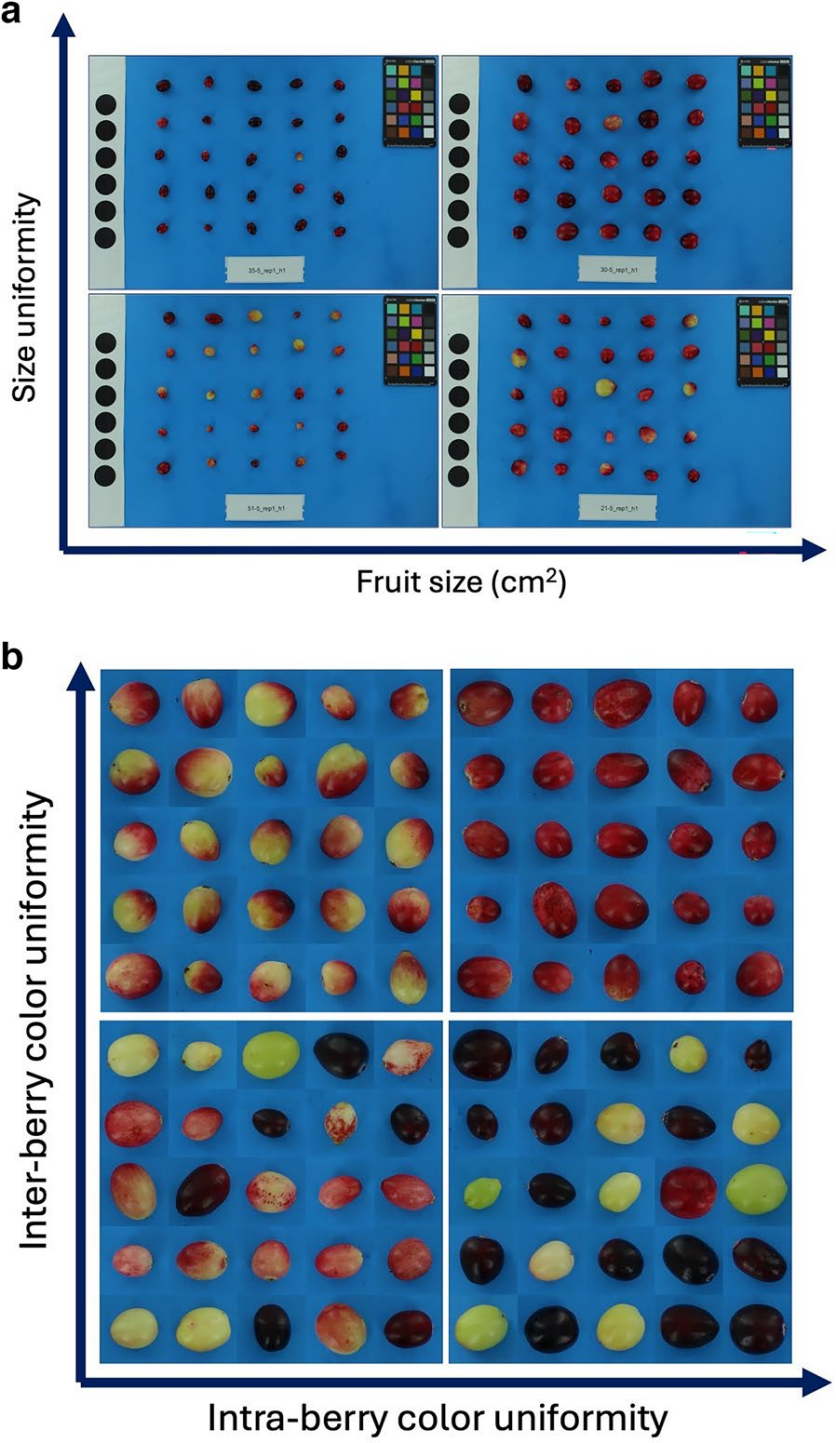
**a** Inter-berry size uniformity and fruit size are important metrics for cranberry processing efficiency. **b** Inter-berry color uniformity and intra-berry color uniformity are important metrics for cranberry processing efficiency

Over 95% of the cranberries produced are machine-harvested. The predominant market for the last 30 years has been the sweetened dried cranberry (SDC) processing market, which has driven the value and demand for high quality cranberries. The most recent influx of cranberry cultivars in the market were bred for color intensity, size and shape, and early fruit maturation [8, 9, 75, 76], which are the highest priority fruit quality traits for processing efficiency by the commercial cranberry industry in the U.S. and Canada [77]. Thus the current cranberry market demands cultivars with inter-berry and intra-berry color and size uniformity (personal communication: Nicole Hansen). One of BerryPortraits’s unique features is its ability to quantify developmental uniformity among berries in a sample, perceptually uniform ripeness in size (Fig. 4a) and color (Fig. 4b), within and between berries. Size uniformity increases harvest efficiency, as mechanical harvesters do not discriminate between ripe and unripe berries. Small berries and berries with incomplete color development are culled during the sorting and packing process. As such, cultivars with a high proportion of uniformly red berries are highly preferred. Furthermore, uniform size increases processing efficiency, as fruit with extreme size variability do not process well during SDC production, and thus are sorted out and culled prior to processing. Identifying breeding lines that produce uniformly sized fruit at harvest will reduce losses for growers and processors. BerryPortraits is also capable of distinguishing between green and white berries, which is important for both breeding and sorting. A fraction of white berries are usable for SDC, as during the infusion step of processing, white berries can absorb the syrup and develop into marketable red or pale red fruit (personal communication: Amaya Atucha). However, green berries become brown after processing, resulting in complete loss of commercial quality. Thus, breeders prioritize advancement of varieties with synchronous ripening and complete color development, which reduces losses for growers due to underdeveloped berries. With the data output from BerryPortraits, breeders can use selection indices to identify, select, and advance cranberry genotypes that meet the needs for cranberry growers and processors (Fig. 4a, b). To this end, selecting genotypes with a high a* value, low a* variance, high L* value, and low L* variance will yield lines that are uniformly red and uniformly bright.

#### Applications

Though developed for plant breeders, BerryPortraits offers innumerable possible research applications for food scientists, plant physiologists, plant pathologists, and horticultural researchers. Repeated measurements over the fruit ripening period to estimate the rate of ripening for different genotypes and identify genotypes suited to seasonal harvest windows, or genotypes with concentrated uniform ripening for machine harvest. Plant geneticists can use this high throughput and accurate phenotype output to identify trait-associated markers in linkage mapping populations or diverse populations. Plant physiologists and horticultural researchers can evaluate correlation of plant pigment accumulation with other morphometric characteristics, or relationships among visual ripening cues and sensory ripening cues. Food science researchers can efficiently and accurately quantify fruit quality under various storage conditions, insect/pathogen damage to fruit, and uniformity metrics provide an updated method for evaluating grades and standards in USDA's agricultural marketing service. The integration of BerryPortraits with BreedBase [59] and Fieldbook [60] enables efficient phenotype data collection.

### Imaging recommendations

Capturing consistent high-quality images is the most important factor in obtaining accurate, reproducible results from BerryPortraits. While a full treatment of photography methods is outside the scope of this paper, below are several basic recommendations for best practices.

Choose a background that is durable and uniform, in a solid color dissimilar to that of the target berries across all stages of fruit ripening and color development. The white background used for some of the images in this study had poor contrast against pale unripe berries, which could cause issues with segmentation. We recommend users test out gray backgrounds available from photography supply vendors. For repeatability and accuracy, the same background should be used for all images you wish to compare or analyze as a single dataset.

Keep image layout consistent, with all berries separated and minimal non-target objects visible in frame. Berries do not need to be aligned into a grid, but this can help with analyzing data and tracing any potential issues.

Always include size markers and a color reference card, ideally a standard X-Rite ColorChecker Classic. Doing appropriate color correction with a proper color reference card is challenging; without one, it is impossible. Similarly, berries cannot be calibrated to real units without appropriate size markers.

Use diffuse lighting from a single controlled light source with a high color rendering index (CRI). Do not rely on ambient daylight, as this can change dramatically within and between days. On berries with reflective epicuticular waxes, direct light without a diffuser or reflector will lead to bright spots of specular reflection, which will throw off color analyses. While diffusers are ideal for waxy berries, reflectors are also useful; either is preferable to direct, non-diffuse light.

When capturing images with a DSLR camera, set white balance before imaging and capture images in RAW format in addition to JPG format. Post hoc color correction on compressed JPG images may be useful, but color correction and white balancing on RAW images is preferable. The ideal protocol is to eliminate or reduce the need for any post hoc color correction by capturing images with a uniform approach.

### Learning and creating in BerryPortraits

Although this program was built to be intuitive and friendly, we encourage users to approach with patience, a growth-mindset, playfulness, and willingness to learn. We advise beginners to acquire some basic familiarity with Python and to continue to grow and enhance their programming skills, which will substantially improve their experience using and creatively building upon BerryPortraits.

Learning a programming language can be both frustrating and rewarding, and thankfully there are abundant free community-based resources for supporting Python learners. Finally, we recommend pilot testing phenotypically representative berry subsamples before launching into a full-scale experimentation.

### Limitations

Though the human health benefits of anthocyanin are well studied and widely known in a variety of crops [8, 9, 78–84], the targeted trait in cranberry breeding programs is the appearance of the color, and the color of interest to the market (bright red) is not as dark or anthocyanin-rich as the darkest berries. BerryPortraits differentiates between bright and dark berries, however researchers may need to troubleshoot to achieve the conditions required to distinguish among the most deeply pigmented berries.

Quantifying berry color is a complex process. Variations in lighting and background can affect mean color values to a drastic extent, and color correction is not a panacea. When comparing two image sets captured under different conditions, any comparison of berry color values is best done with a high degree of caution.

It is not currently known how these visual ripeness traits correlate with indicators of ripeness (such as degrees Brix, volatiles, or other physiological ripeness traits). An important caveat in our grower-friendly definition of “ripeness” is that fully physiologically ripe fruit can have incomplete (non-red) color development.

Finally, we advise that researchers of climacteric fruit minimize the time between harvest and imaging, as fruit color may continue to develop and change in the interim.

## Conclusions

BerryPortraits is an open source, Python-based, high throughput phenotyping platform that accurately measures berry color, size, and shape, saving an abundance of time, labor, and cost in horticultural research programs. There are innumerable possible creative applications for this software by food scientists, plant physiologists, horticultural researchers, and plant breeders. BerryPortraits has strong potential to be adapted for phenotyping other berry crops, such as grape, blueberry, lingonberry, caneberry, and any crops in which color, size, shape, and uniformity are important for fruit quality, production, or marketability.

## Supplementary Information

Supplementary Material 1. Supp. Fig. 1: Cranberry samples from a single genotypestaged within an enclosed lightbox on a blue background with black 2.54-cm scale markers, color card, and sample label. Camera is placed atop the lightbox and the lens dropped through an opening in the lightbox’s ceiling. Black light curtain with reflective interior is replaced over lightbox before image capture. The imaging field is displayed on the computer monitor. A homemade wooden block with 5x5 bored holes facilitates rapid berry placement. Supp. Fig. 2: Example of berry imaging photographs inside the enclosed light box for controlled and standardized lighting conditions. Sample of background 30 fruit with 2.54-cm diameter black scale markers. Photograph from digital SLR. Supp. Fig. 3: Examples of cranberry images used for training set with YOLOv8. Supp. Fig. 4: Volumeapproximated as a series of stacked cylinders, 1 pixel high; surface areaapproximated as a series of stacked truncated cones, 1 pixel high. Supp. Fig. 5: Solidity comparison between BerryPortraits and GiNA. BerryPortraits had fewer berries below the 0.95 threshold than GiNA . While both platforms demonstrate excellent ability to segment fruit, BerryPortraits has higher overall performance for solidity. GiNA’s segmenting ability appears to decrease with pink or light red fruit, while in Berry Portraits, solidity appears to decrease with yellow fruit. Supp. Fig. 6: Range of colors in cranberry population under study. L*a*b*values converted to hexcode for visualization

## Data Availability

BerryPortraits software is available at https://github.com/Breeding-Insight/BerryPortraits/. The datasets used and/or images analyzed are available at https://cggl.horticulture.wisc.edu/ or from Juan Zalapa (juan.zalapa@usda.gov) on reasonable request. Availability and requirements: Project name: BerryPortraits: Phenotyping Of Ripening Traits with Rapid Automated Imaging Tools. Project home page: https://github.com/Breeding-Insight/BerryPortraits/. Operating system(s): Platform independent. Programming language: Python. Other requirements: Users of BerryPortraits web app will require an ORCID ID for authentication. Users of BerryPortraits code will require a GitHub account to fork or download the code.

## Abbreviations

CIELAB: Perceptually uniform color space that expresses color as three values based on human perception from the visible spectrum: L*a*b*
CIE (from CIELAB): *Commission Internationale de l’Eclairage*
L* (from CIELAB): L* for luminance (perceptual lightness)
a* (from CIELAB): A* for red/green color coordinates
b* (from CIELAB): B* for blue/yellow color coordinates
DSLR: Digital single lens reflex (camera)
MLP: Multilayer perceptron
QTL: Quantitative trait locus/loci
RGB: Red/green/blue channel
SDC: Sweetened dried cranberries
TAcy: Total anthocyanin
YOLOv8: You Only Look Once (version 8)
