# Correction: BerryPortraits: phenotyping of ripening traits in cranberry (Vaccinium macrocarpon Ait.) With YOLOv8

**DOI:** 10.1186/s13007-025-01323-6

**Published:** 2025-01-07

**Authors:** Jenyne Loarca, Tyr Wiesner-Hanks, Hector Lopez-Moreno, Andrew F. Maule, Michael Liou, Maria Alejandra Torres-Meraz, Luis Diaz-Garcia, Jennifer Johnson-Cicalese, Jeffrey Neyhart, James Polashock, Gina M. Sideli, Christopher F. Strock, Craig T. Beil, Moira J. Sheehan, Massimo Iorizzo, Amaya Atucha, Juan Zalapa

**Affiliations:** 1 Department of Plant and Agroecosystem Sciences, University of Wisconsin-Madison, Madison, WI USA; 2 United States Department of Agriculture-Agricultural Research Service, Vegetable Crops Research Unit, Madison, WI USA; 3 Cornell University-Breeding Insight, Ithaca, NY USA; 4 Department of Statistics, University of Wisconsin-Madison, Madison, WI USA; 5 Department of Viticulture and Enology, University of California-Davis, Davis, CA USA; 6 Phillip E. Marucci Center for Blueberry and Cranberry Research & Extension, Chatsworth, NJ USA; 7 United States Department of Agriculture-Agricultural Research Service, Genetic Improvement of Fruits & Vegetables Laboratory, Beltsville, MD USA; 8 Rutgers University-Department of Plant Biology, New Brunswick, NJ USA; 9 Department of Horticultural Science, North Carolina State University, Raleigh, NC USA; 10 Plant Human Health Institute, North Carolina State University, Raleigh, NC USA

**Correction: Plant Methods (2024) 20:172**

10.1186/s13007-024-01285-1

In this article the title was incorrectly given as ‘BerryPortraits: Phenotyping Of Ripening Traits cranberry (Vaccinium macrocarpon Ait.) with YOLOv8’.

but should have been.

‘BerryPortraits: Phenotyping Of Ripening Traits **in** cranberry (Vaccinium macrocarpon Ait.) with YOLOv8’.

The authors would like to include following sentence in the Acknowledgements.

“The authors thank Collins Wakholi and Devin Rippner for their generous consultation and sharing their knowledge of digital image color correction.”

The sentence currently reads:

The authors would like to extend gratitude to Nicholi Vorsa for his care in cre ating and stewarding the CNJ02-1 population and for sharing propagules for establishment in Wisconsin; AJ Ackerman, Josue Chinchilla-Vargas, Edwin Rei del, and Tyler Slonecki (and the entire Breeding Insight science team); David Meidlinger, (and the entire Breeding Insight development team); the BI leadership, program coordinators, and technical steering board; Jim Spoden, Kristy Morrow, Ree Klumb, Randey Lewis, Gustavo Ivan Hernandez-Sierra, and Eric Wiesman for their diligent assistance in the lab; and Nicole Hansen and Bill Hatch (Cranberry Creek Inc.) for efusively sharing their knowledge of the commercial cranberry industry, which helped us to identify relevant traits for cranberry growers. JZ would like to express his gratitude through PS 136:1.

The sentence should read:

The authors would like to extend gratitude to Nicholi Vorsa for his care in cre ating and stewarding the CNJ02-1 population and for sharing propagules for establishment in Wisconsin; AJ Ackerman, Josue Chinchilla-Vargas, Edwin Rei del, and Tyler Slonecki (and the entire Breeding Insight science team); David Meidlinger, (and the entire Breeding Insight development team); the BI leadership, program coordinators, and technical steering board; Jim Spoden, Kristy Morrow, Ree Klumb, Randey Lewis, Gustavo Ivan Hernandez-Sierra, and Eric Wiesman for their diligent assistance in the lab; and Nicole Hansen and Bill Hatch (Cranberry Creek Inc.) for efusively sharing their knowledge of the commercial cranberry industry, which helped us to identify relevant traits for cranberry growers. JZ would like to express his gratitude through PS 136:1.

“The authors thank Collins Wakholi and Devin Rippner for their generous consultation and sharing their knowledge of digital image color correction.”

The original article has been corrected.

