# Supplementary figures and images for: BerryPortraits: Phenotyping Of Ripening Traits in cranberry (Vaccinium macrocarpon Ait.) with YOLOv8
Source: Plant Methods. 2024 Nov 13;20:172. doi: 10.1186/s13007-024-01285-1 (PMC11562335; doi:10.1186/s13007-024-01285-1)

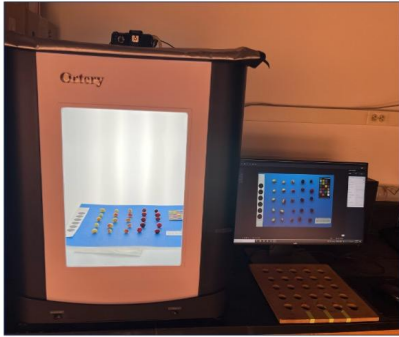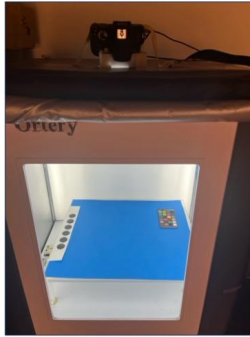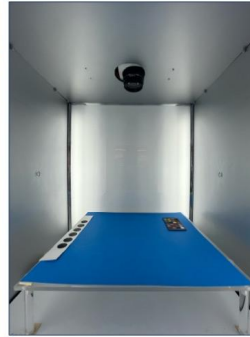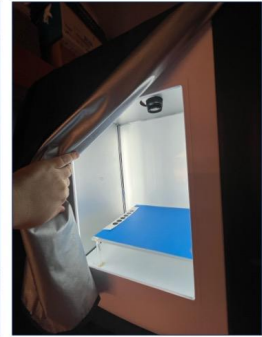

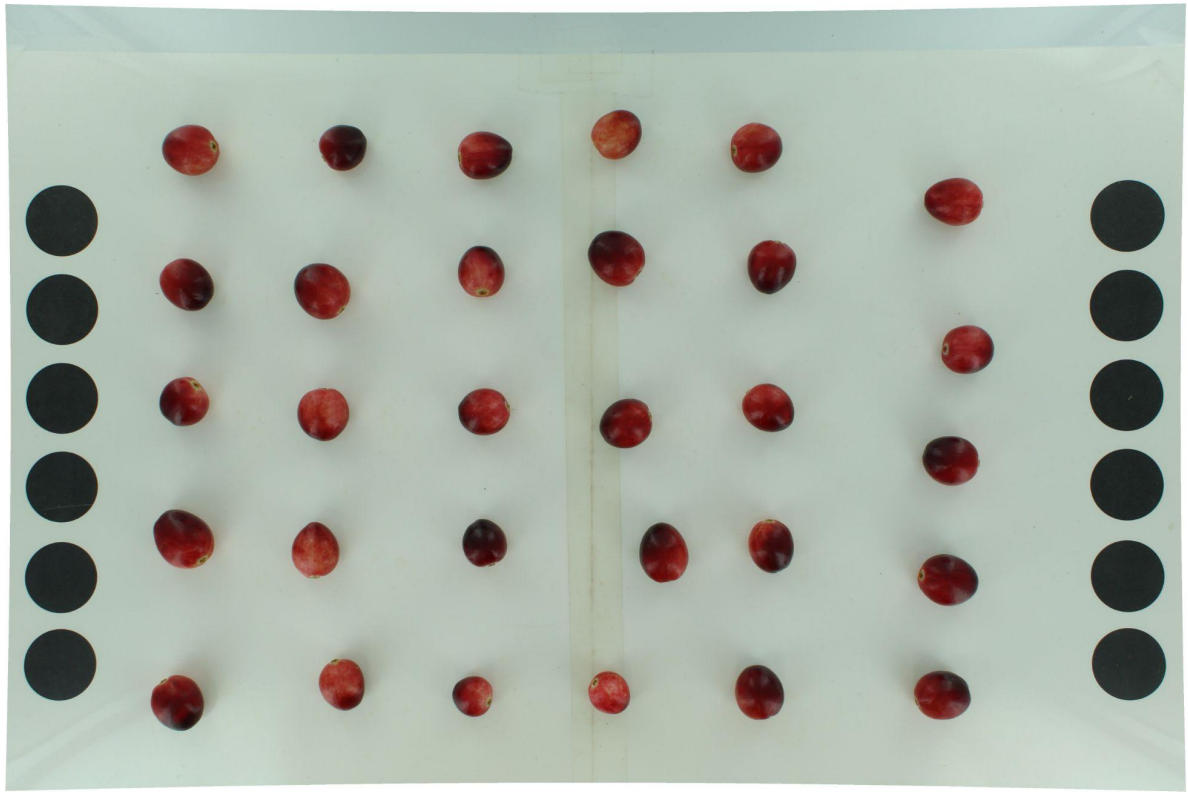

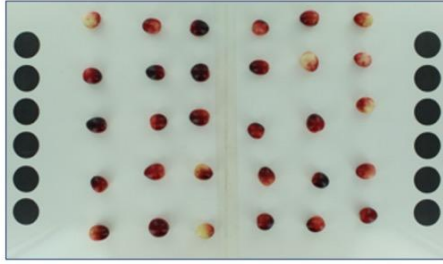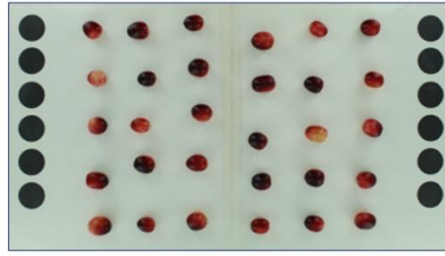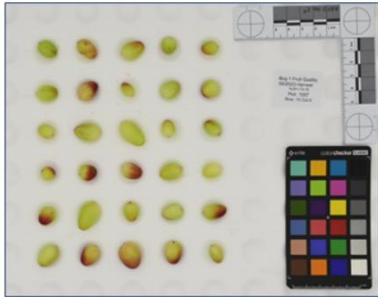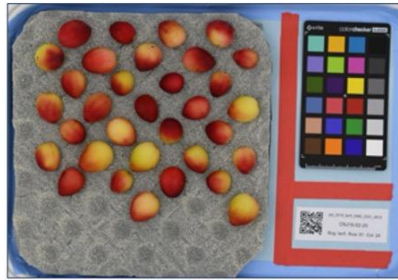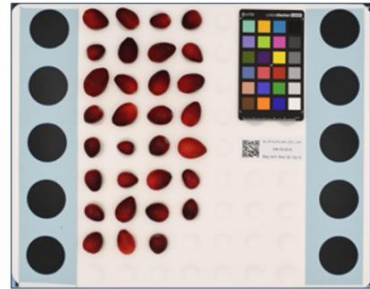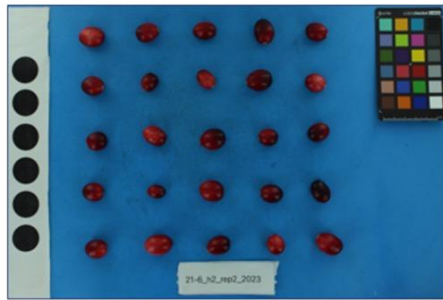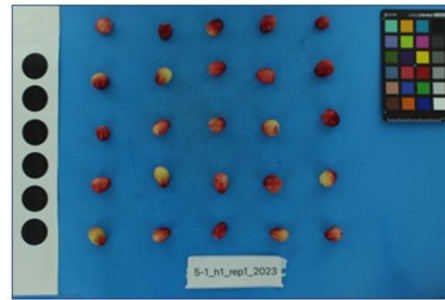

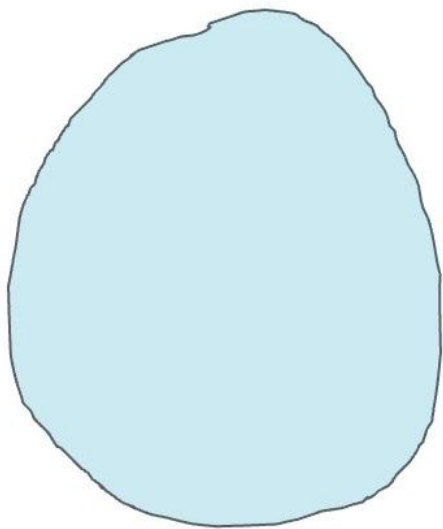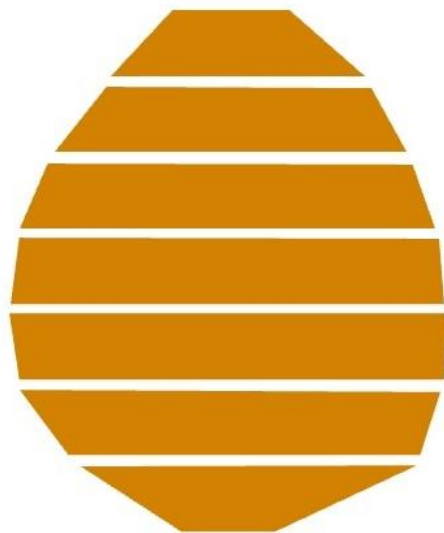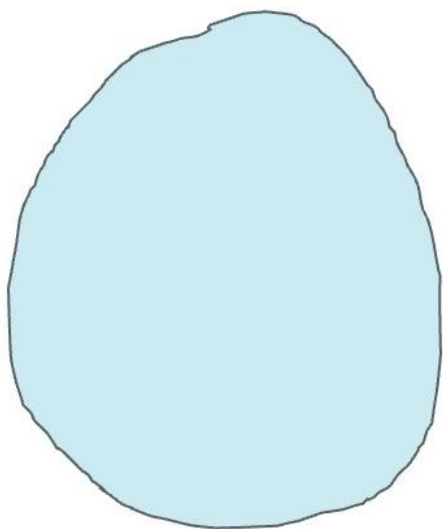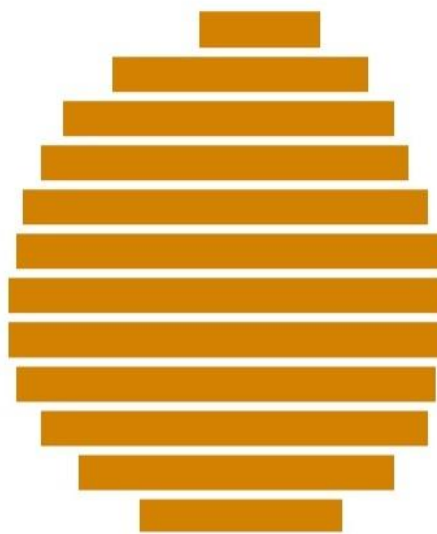

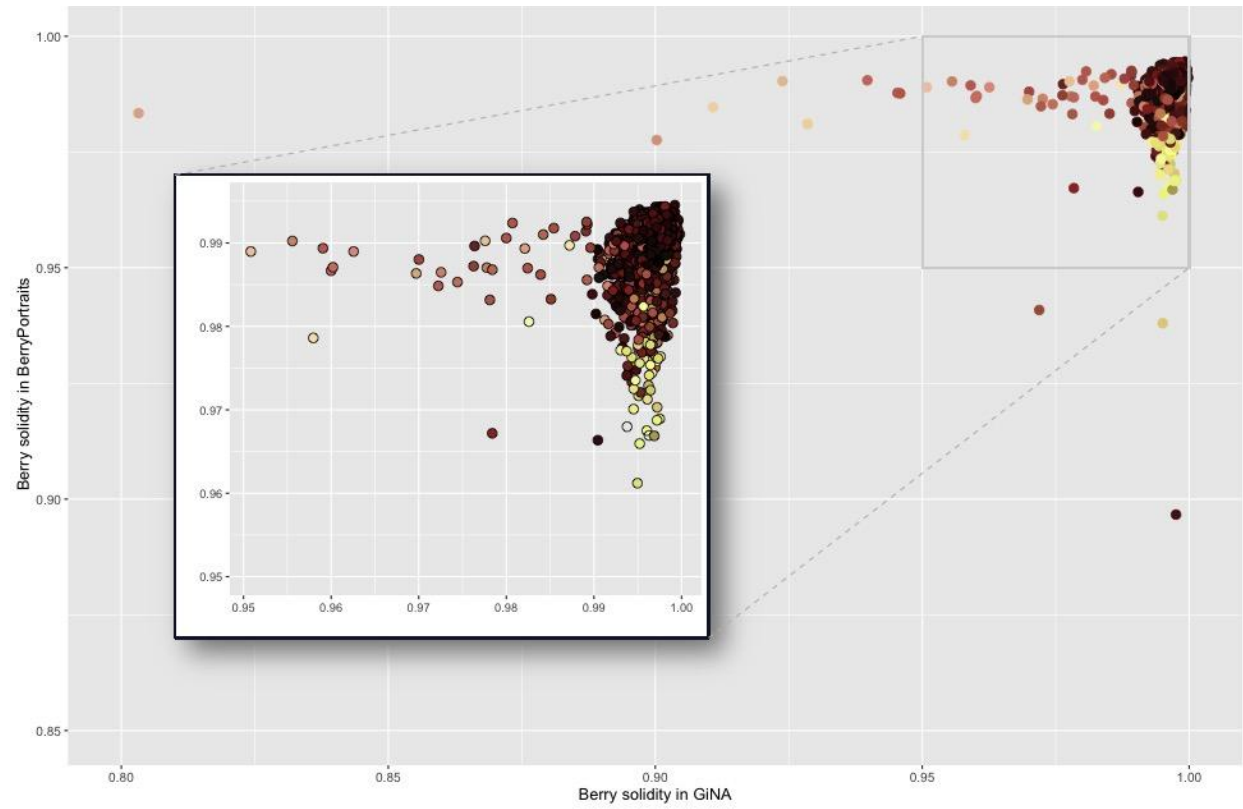

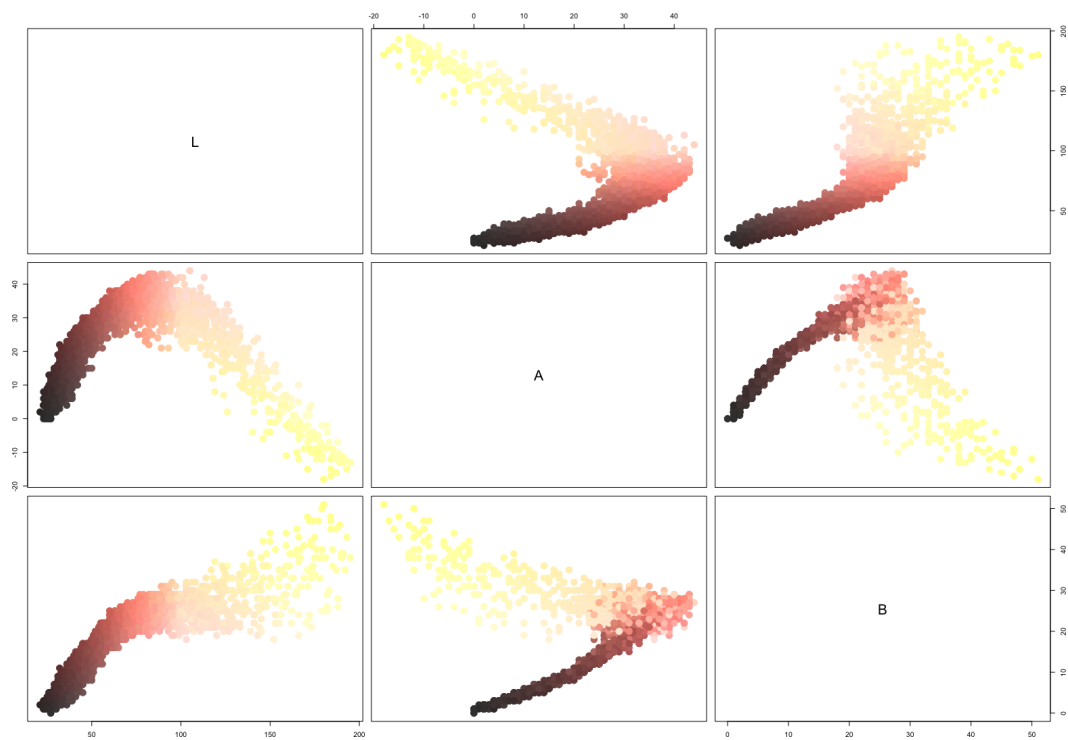

|  |  |  |  |  |  |
|--|--|--|--|--|--|
|  |  |  |  |  |  |
|  |  |  |  |  |  |
|  |  |  |  |  |  |
|  |  |  |  |  |  |
|  |  |  |  |  |  |

[illegible]

Supplement: Supplementary file 1 — Supplementary Material 1. Supp. Fig. 1: Cranberry samples from a single genotypestaged within an enclosed lightbox on a blue background with black 2.54-cm scale markers, color card, and sample label. Camera is placed atop the lightbox and the lens dropped through an opening in the lightbox’s ceiling. Black light curtain with reflective interior is replaced over lightbox before image capture. The imaging field is displayed on the computer monitor. A homemade wooden block with 5x5 bored holes facilitates rapid berry placement. Supp. Fig. 2: Example of berry imaging photographs inside the enclosed light box for controlled and standardized lighting conditions. Sample of background 30 fruit with 2.54-cm diameter black scale markers. Photograph from digital SLR. Supp. Fig. 3: Examples of cranberry images used for training set with YOLOv8. Supp. Fig. 4: Volumeapproximated as a series of stacked cylinders, 1 pixel high; surface areaapproximated as a series of stacked truncated cones, 1 pixel high. Supp. Fig. 5: Solidity comparison between BerryPortraits and GiNA. BerryPortraits had fewer berries below the 0.95 threshold than GiNA . While both platforms demonstrate excellent ability to segment fruit, BerryPortraits has higher overall performance for solidity. GiNA’s segmenting ability appears to decrease with pink or light red fruit, while in Berry Portraits, solidity appears to decrease with yellow fruit. Supp. Fig. 6: Range of colors in cranberry population under study. L*a*b*values converted to hexcode for visualization [file 13007_2024_1285_MOESM1_ESM.pdf]
